# High-Frequency Binaural Beats Increase Cognitive Flexibility: Evidence from Dual-Task Crosstalk

**DOI:** 10.3389/fpsyg.2016.01287

**Published:** 2016-08-24

**Authors:** Bernhard Hommel, Roberta Sellaro, Rico Fischer, Saskia Borg, Lorenza S. Colzato

**Affiliations:** ^1^Institute for Psychological Research, Leiden Institute for Brain and Cognition, Leiden UniversityLeiden, Netherlands; ^2^Department of Psychology, University of GreifswaldGreifswald, Germany

**Keywords:** PRP, Dual-task, Binaural beats, gamma

## Abstract

Increasing evidence suggests that cognitive-control processes can be configured to optimize either persistence of information processing (by amplifying competition between decision-making alternatives and top-down biasing of this competition) or flexibility (by dampening competition and biasing). We investigated whether high-frequency binaural beats, an auditory illusion suspected to act as a cognitive enhancer, have an impact on cognitive-control configuration. We hypothesized that binaural beats in the gamma range bias the cognitive-control style toward flexibility, which in turn should increase the crosstalk between tasks in a dual-task paradigm. We replicated earlier findings that the reaction time in the first-performed task is sensitive to the compatibility between the responses in the first and the second task—an indication of crosstalk. As predicted, exposing participants to binaural beats in the gamma range increased this effect as compared to a control condition in which participants were exposed to a continuous tone of 340 Hz. These findings provide converging evidence that the cognitive-control style can be systematically biased by inducing particular internal states; that high-frequency binaural beats bias the control style toward more flexibility; and that different styles are implemented by changing the strength of local competition and top-down bias.

## Introduction

The concept of cognitive control refers to processes that are not directly involved in processing and selecting stimulus events or actions but that rather orchestrate the processes responsible for these basic functions. Control processes are commonly characterized in terms of their capacity limitations but there is increasing evidence that they can also vary in style. Both functional (Goschke, 2003; Dreisbach and Goschke, 2004) and neural (Cools, 2008, 2012; Cools and D’Esposito, 2011) considerations suggest that cognitive-control states can vary both intra- and inter-individually to the degree that they either focus the available processing capacity on one single event or task or distribute capacity more widely across various processes or tasks. Following these leads, Hommel (2015) has suggested that the style of control varies between persistence and flexibility: while the former implies highly focused, exclusive processing, the latter implies a broad distribution of resources and rather integrative processing. As the current settings on the persistence-flexibility dimension affect both the basic cognitive operations and the operation characteristics of the superordinate cognitive-control processes, Hommel (2015) refers to the process of adjusting and changing the settings as “metacontrol” and to the resulting settings or states as “metacontrol states.”

There are increasing attempts to identify means to bias metacontrol states in systematic ways, which is of both theoretical and practical relevance. It is of theoretical relevance because the characterization of effective means to bias the metacontrol state or control style provides constrains for understanding its underlying functional and neural mechanisms. And it is of practical relevance because effective means point to interesting methods for individually tailored cognitive enhancement, which for instance might seek to support individuals to implement particularly adaptive states according to their needs. The present study assessed an enhancement technique that has been frequently claimed to target cognitive control functions: binaural beats—the subjective experience of a beating tone with a frequency that corresponds to the frequency difference between two binaurally presented tones (Oster, 1973). Originally, binaural beats of low frequency have been argued to induce mental relaxation while high frequencies were assumed to induce alertness and attentional concentration (Vernon, 2009; Turow and Lane, 2011). This would suggest that high-frequency beats bias cognitive control toward persistence and focus, but recent findings suggest the exact opposite.

In a recent study, we presented participants with high-frequency binaural beats (gamma range), low-frequency binaural beats (alpha range), or a continuous tone of 340 Hz (Reedijk et al., 2015) before they performed an attentional blink task (Raymond et al., 1992). In this task, participants are presented with two visual targets in a rapid stream of stimuli, which commonly leads to the observation that they often miss the second target if it is presented briefly after the first. The impact of the low-frequency beats on the attentional blink did not differ from the control condition, while the high-frequency beats reduced the attentional blink significantly in individuals with low striatal dopamine. The presence of the attentional blink has been attributed to over-control (Olivers and Nieuwenhuis, 2006)—i.e., a too strong focus on the first target, which leaves too few resources for the second. This suggests that high-frequency beats lead to a broader distribution, rather than to a stronger focus, of available resources—to more cognitive flexibility that is. This interpretation would fit the observation that binaural beats in the gamma range can improve performance in a divergent thinking task, but not in a convergent thinking task (Reedijk et al., 2013), as divergent thinking should benefit more from broadly distributed resources than convergent thinking.

The present study sought for converging evidence for the idea that binaural beats in the gamma range might bias cognitive control toward flexibility. In previous studies, control biases toward persistence or flexibility have been assessed by means of crosstalk between different event representations or across multiple tasks (e.g., Dreisbach and Goschke, 2004). Of particular relevance for our present study, Fischer and Hommel (2012) have tested participants in a dual-task paradigm after having primed them with a convergent-thinking task or a divergent-thinking task. The dual-task paradigm was chosen to produce the well-established psychological refractory period (PRP) effect (see Pashler, 1998, for an overview): the observation that a response (R2) to a stimulus (S2) is slower the sooner this stimulus appears after the presentation of another stimulus (S1) signaling another response (R1). In other words, the reaction time for the second of two responses (RT2) increases as the interval between S1 and S2 (the stimulus onset asynchrony or SOA) decreases. The idea was that a convergent or divergent priming task would bias the control style toward persistence versus flexibility, respectively. The dependent measure of interest was the degree of crosstalk from the second on the first task. As previously demonstrated, RT1 (the reaction time in the first-performed task) is sensitive to the compatibility between the response in the first task (R1) and the response in the second (R2: Hommel, 1998; Logan and Schulkind, 2000); for instance, the time it takes to press the left of two keys in the first task (R1) is faster if the second task also requires a left keypress (R2). This demonstrates that R2 is activated before R1 selection is completed, which makes the response-compatibility effect (RCE) an indicator of the degree of distributed, parallel processing (Logan and Gordon, 2001; Lien and Proctor, 2002).

As one would expect from this reasoning, Fischer and Hommel (2012) found a smaller RCE if participants were primed with a convergent-thinking rather than a divergent-thinking task. If we assume that engaging in divergent thinking leads to a more broadly distributed allocation of processing resources, and that this bias toward more flexibility was sufficiently inert to affect performance in the overlapping dual task, we can conclude that the size of the RCE reflects the relative bias toward persistence and flexibility. If our hypothesis that high-frequency binaural beats bias the cognitive control style toward flexibility is correct, presenting participants with high-frequency beats should thus increase their RCE in a dual task that manipulates R1-R2 compatibility. We tested this prediction by adopting a task comparable to that used by Fischer and Hommel (2012) and having participants perform it after presenting them with either high-frequency binaural beats (the gamma group) or with a continuous tone of 340 Hz (the control group). Given that binaural beats may impact mood (Chaieb et al., 2015), heart rate, and human blood pressure (Carter, 2008), we also assessed participants’ subjective affective states, heart rate, and blood pressure before and after the dual-task performance.

## Materials and Methods

### Participants

Forty students (32 female, eight male; aged 18–27 years old) from Leiden University took part in exchange for course credit or pay. All had normal or corrected-to-normal sight and hearing. Participants were selected individually using the Mini International Neuropsychiatric Interview (M.I.N.I.; Sheehan et al., 1998), a well-established brief diagnostic tool in clinical, drug and stress research that screens for several psychiatric disorders and drug use (Sheehan et al., 1998; Colzato and Hommel, 2008; Colzato et al., 2008). A group of randomly selected 20 participants (15 female, five male) was exposed to gamma-frequency (40 Hz) binaural beats and the other 20 (17 female, three male) were assigned to a control condition, in which they were exposed to a constant tone of 340 Hz.

### Ethical Statement

Written informed consent was obtained from all subjects; the protocol and the remuneration arrangements of 5 euro were approved by the local ethical committee (Leiden University, Institute for Psychological Research).

### Dual-Task Paradigm

Like Fischer and Hommel (2012), we adopted the dual-task paradigm from Fischer et al. (2007; see also Logan and Schulkind, 2000), in which both tasks required participants to categorize the stimuli as being smaller vs. larger than 5. To avoid identical stimuli in both tasks (e.g., perceptual match) and to maintain the numerical distance to 5, the digits 3 and 7 and digits 2, 4, 6, and 8, presented in white on black background, served as stimuli for Task 1 (S1) and Task 2 (S2), respectively (Fischer et al., 2007). The same categorization of S1 and S2 (i.e., either both smaller or both larger than 5) implied a match between R1 and R2 categories, that is, response-category compatibility or response compatibility for short. Accordingly, opposite categorizations (i.e., S1 smaller and S2 larger than 5 or vice versa) implied response incompatibility, so that performance differences between response-compatible and response-incompatible trials reflect the RCE.

Participants were to press one of two keys in each task: the “,” and “.” key of the QWERTZ keyboard to S1, by using their right index and middle finger, and the “Y” and the “X” key to S2, by using their left middle and index finger. The stimulus-response mappings were counterbalanced across participants. Each trial began with a 500-ms fixation display, next to which S1 appeared above the screen center. Following an SOA of 40, 130, 300, or 900 ms, S2 appeared below screen center for 1000 ms. Both stimuli were replaced by a 2-s blank screen, followed by the 300 ms feedback “correct” or, in case of an incorrect response in either task, a missing response, or incorrect response order, the feedback “error.” Participants were asked not to group responses and to respond as fast and as accurately as possible, first to S1 and only second to S2 (Task 1 priority). Participants performed 64 practice trials, followed by three experimental blocks of 64 trials each.

### Procedure

Participants were tested individually. Upon arrival, they were asked to rate their mood on a 9 × 9 Pleasure × Arousal grid (Russell et al., 1989), with values ranging from –4 to 4. The resulting score thus indicated the location of the participant’s affective state within a two-dimensional space defined by hedonic tone and activation. Subsequently, participants listened to gamma-frequency (40 Hz) binaural beats or a constant tone of 340 Hz (control condition), all embedded in white noise to enhance clarity of the beats (Oster, 1973), for 3 min before and during the dual-task paradigm (training and experimental blocks). Binaural beats were presented through in-ear headphones (Etymotic Research ER-4B microPro), which provide 35 dB noise attenuation. The binaural beats were based on a 340 Hz carrier tone, which was used as the constant tone in the control condition. After the dual-task paradigm, participants rated their mood for the second time. After these measurements the experimental session ended and participants were paid, debriefed, and dismissed.

### Statistical Analyses

In view of the relatively small number of trials in each design cell we did not trim the data but analyzed median rather than mean RTs to reduce the impact of outliers. To assess whether binaural beats modulate dual-task performance, median RTs data for T1 and T2 were submitted to two separate repeated-measures analyses of variance (ANOVAs) with SOA (40 vs. 130 vs. 300 vs. 900) and Response Compatibility (R1-R2 compatible vs. R1-R2 incompatible) as within-participants factors and group (control vs. gamma) as between-participants factor.

Incorrect T1 (*M* = 1.4%, *SEM* = 0.2) and T2 responses (*M* = 4.2%, *SEM* = 0.6) were excluded and the analyses were restricted to trials in which both responses were correct. Mood (pleasure and arousal scores), heart rate (HR), systolic blood pressure (SBP) and dystolic blood pressure (DBP) were analyzed separately by means of repeated-measures ANOVAs. Effect of time (first vs. second measurement) served as within-subjects factor and group (gamma vs. control) as between-subject factor. A significance level of *p* < 0.05 was adopted for all statistical tests. In case of significant interaction, *post hoc* analyses were conducted using Tukey HSD test.

## Results

### Participants

No significant group differences were observed in terms of age (*M* = 19.8, *SEM* = 0.6 and *M* = 20.25, *SEM* = 0.7 for the control and gamma group, respectively), *t*(38) < 1, *p* = 0.64, or gender distribution (F/M = 17/3 and 15/5 for the control and gamma group, respectively), χ2 < 1, *p* = 0.43.

### RT1

The main effect of SOA was significant, *F*(3,114) = 10.524, *p* < 0.001, η_p_^2^ = 0.22, indicating faster RTs with increasing SOA. *Post hoc* analyses showed that RTs were significantly slower at SOA-40 and SOA-130 than at SOA-300 and SOA-900 (*p*_s_ ≤ 0.007). No significant differences were observed between SOA-40 and SOA-130 (*p* = 0.999), or between SOA-300 and SOA-900 (*p* = 0.62).

**FIGURE 1:**
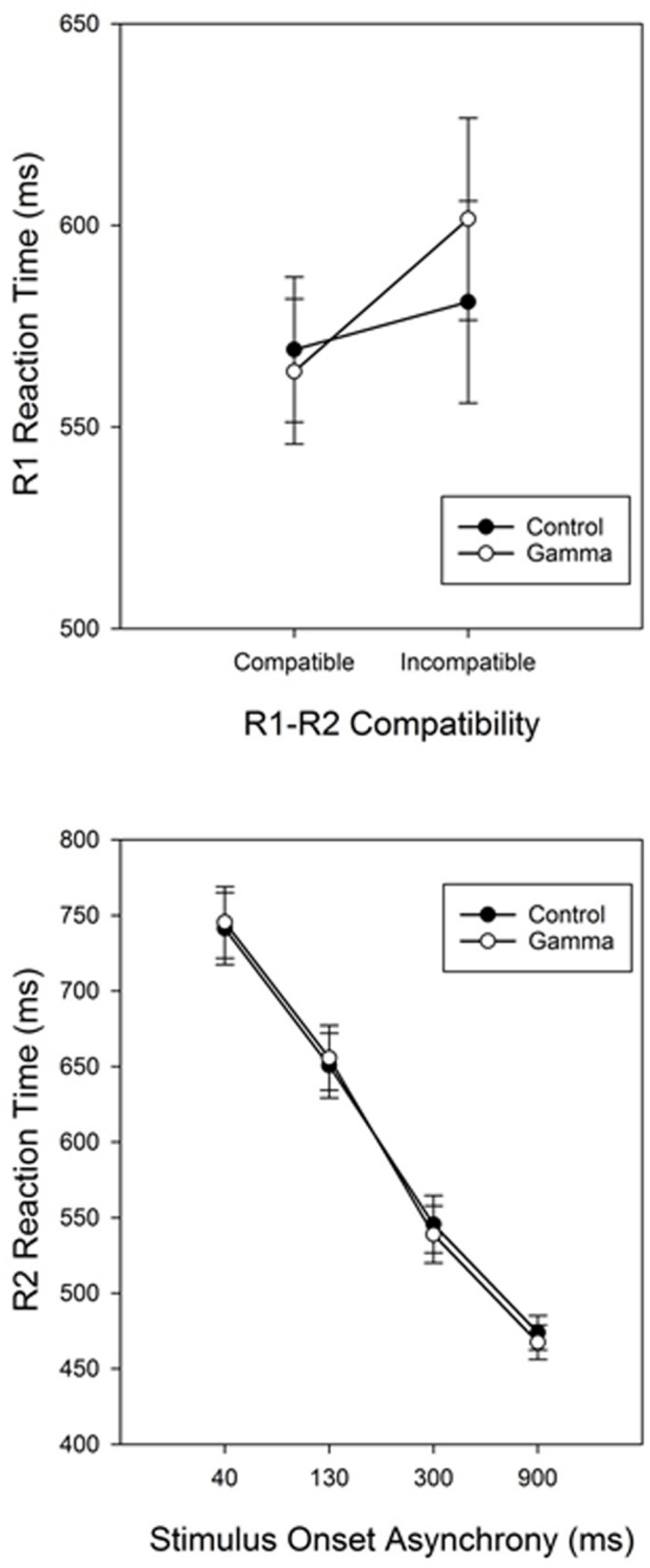
**Response-compatibility (R1-R2 Compatibility) effect in Task 1 (RT1) for the control and gamma groups (top panel).** Reaction times for Task 2 (RT2) as a function of group (control vs. gamma) and stimulus onset asynchrony (bottom panel). Error bars represent standard errors of the response-compatibility effect (Task 1) and the PRP effect (Task 2).

The main effect of Response Compatibility was also significant, *F*(1,38) = 15.799, *p* < 0.001, η_p_^2^ = 0.29, with participants being faster in categorizing S1 when S1 and S2 belonged to the same response category (*M* = 566, *SEM* = 12.7) than when they did not (*M* = 591, *SEM* = 17.7) (see **Figure 1** and **Table 1**). This effect was significant for short SOAs only (68 and 43 ms for SOA-40 and SOA-130, respectively, *p*_s_ < 0.001), but not for long SOAs (12 and 0 ms, for SOA-300 and SOA-900, respectively, *p*_s_ ≥ 0.88), as revealed by a significant interaction between SOA and Response Compatibility, *F*(3,114) = 17.78, *p* < 0.001, η_p_^2^ = 0.32. More importantly, we observed a significant interaction between group and Response Compatibility, *F*(1,38) = 4.33, *p* = 0.04, η_p_^2^ = 0.10, showing that the compatibility effect was significantly (and more than three times) larger in the gamma group (38 ms) than in the control group (12 ms). No other significant effects were found, *F*_s_ ≤ 1.79, *p*_s_ ≥ 0.15.

**Table 1:** Reaction times (in ms) for Task 1 (RT1) and Task 2 (RT2) as a function of group (control and gamma), response-category compatibility (R1-R2 compatibility), and stimulus onset asynchrony (SOA).

Task	Group	R1-R2 compatibility	SOA			
			40	130	300	900
**RT1**	Control	Compatible	560 (14.8)	573 (17.6)	585 (25.4)	559 (20.9)
		Incompatible	610 (30.2)	598 (25.6)	566 (26.1)	551 (24.4)
			50	25	-19	-8
	Gamma	Compatible	561 (14.8)	573 (17.6)	564 (25.4)	557 (20.9)
		Incompatible	647 (30.2)	634 (25.6)	559 (26.1)	566 (24.0)
			87	60	-5	9
**RT2**	Control	Compatible	668 (17.0)	605 (18.1)	527 (19.1)	468 (12.5)
		Incompatible	815 (33.9)	696 (26.6)	564 (20.4)	480 (11.4)
			147	91	37	12
	Gamma	Compatible	663 (17.0)	595 (18.1)	513 (19.1)	462 (12.5)
		Incompatible	827 (33.9)	716 (26.6)	565 (20.4)	473 (11.4)
			164	121	52	11

*Standard errors of the means are presented in parenthesis.*

### RT2

The main effect of SOA was significant, *F*(3,114) = 450.126, *p* < 0.001, η_p_^2^ = 0.92, reflecting the typical PRP effect with steeply increasing reaction times as SOAs get shorter (Pashler, 1994). *Post hoc* analyses revealed significant differences between all SOAs, *p*_s_ < 0.001. The main effect of Response Compatibility was significant too, *F*(1,38) = 90.394, *p* < 0.001, η_p_^2^ = 0.70, indicating faster RTs for compatible trials (*M* = 563, *SEM* = 10.9) than for incompatible trials (*M* = 642, *SEM* = 15.7). This effect varied as a function of SOA, as indicated by a significant Response Compatibility × SOA interaction, *F*(3,114) = 50.681, *p* < 0.001, η_p_^2^ = 0.57. *Post hoc* analyses revealed that the RCE was significant for SOAs 40, 130, and 300 (155, 104, and 45 ms, respectively, *p*_s_ < 0.001), but not for the SOA-900 (11 ms, *p* = 0.91). There was no other significant effect, *F*_s_ ≤ 1.79, *p*_s_ ≥ 0.36.

### Physiological and Mood Measurements

ANOVAs showed a main effect of time for pleasure, *F*(1,38) = 8.792, *p* = 0.005, η_p_^2^ = 0.19, arousal *F*(1,38) = 11.868, *p* = 0.001, η_p_^2^ = 0.24, and HR, *F*(1,38) = 9.727, *p* = 0.003, η_p_^2^ = 0.20, but not for SBP and DBP, *F*_s_ < 1, *p*_s_ ≥ 0.43. Pleasure, arousal and HR levels decreased during the experiment [Pleasure: *M*_Time 1_ = 1.6 (*SEM*_Time 1_ = 0.2) vs. *M*_Time 2_ = 1.1 (*SEM*_Time 2_ = 0.2); Arousal: 0.6 (0.2) vs. -0.4 (0.3); HR: 86.0 (2.4) vs. 77.5 (2.3)], whereas SBP [124.0 (2.4) vs. 122.5 (2.8)] and DPB [72.4 (1.4) vs. 72.8 (2.0)] did not vary across time. Importantly, neither the group effect nor the interaction was significant, *F*_s_ ≤ 2.6, *p*_s_ ≥ 0.11, suggesting that physiological and mood changes were comparable across groups: Pleasure [Control: 1.4 (0.2) vs. 1.1 (0.3); Gamma: 1.9 (0.2) vs. 1.1 (0.3)], arousal [Control: 0.5 (0.3) vs. -0.7 (0.4); Gamma: 0.7 (0.3) vs. -0.1 (0.4)], HR [Control: 89.3 (3.4) vs. 79.8 (3.3); Gamma: 82.7 (3.4) vs. 75.2 (3.3)], SBP [Control: 120.3 (3.4) vs. 120.3 (3.9); Gamma: 127.8 (3.4) vs. 124.8 (3.9)] and DBP [Control: 71.5 (2.0) vs. 74.8 (2.8); Gamma: 73.2 (2.0) vs. 70.9 (2.8)]. This suggests that we can rule out an account of our results in terms of physiological and/or mood changes.

## Discussion

We tested the possibility that high-frequency binaural beats in the gamma range bias cognitive control toward more flexibility. We hypothesized that this would induce more crosstalk between the two tasks in a dual-task paradigm, resulting in a more pronounced RCE in the first task after being exposed to gamma beats than in a control condition. The findings show the predicted result and there was no indication that mood or other physiological changes were responsible for, or related to this effect (even though we acknowledge that a possible moderation by mood need not be inconsistent with our prediction, as both cognitive control and mood rely on dopaminergic supply and are thus sensitive to changes therein: e.g., Akbari Chermahini and Hommel, 2012). We thus consider the present findings to support the assumption that gamma beats promote cognitive flexibility. This has both theoretical and practical relevance, as it shows that control states can be affected and be systematically biased by task-irrelevant stimulation. This seems to suggest that cognitive-control states can be triggered exogenously, which challenges the traditional idea that stimulus processing and response selection emerges from the competition between endogenous control operations and exogenous, stimulus-induced tendencies (e.g., Verbruggen et al., 2014). On the positive side, our findings suggest that binaural beats provide the opportunity for cognitive enhancement by providing people with tools to tailor their cognitive-control states to situational demands. In particular, binaural beats seem to provide the opportunity to increase people’s cognitive flexibility in a rather automatic fashion, that is, without any particular instruction or task-relevance of the beats. We note that our sample is predominantly female, a common limitation for studies using psychology students as participants. On the one hand, the two experimental groups were matched for gender, so that this general gender imbalance cannot account for our main findings. On the other hand, however, more research will be necessary to see whether these findings generalize to males.

Before speculating on the possible neural mechanisms underlying the impact of binaural beats, we would like to discuss a recent finding that does not seem to fit with our flexibility hypothesis. In particular, Colzato et al. (2015) observed that binaural gamma beats reduced the global-precedence effect in a Navon task (i.e., better performance to the global than to the local features of a visual stimulus) and interpreted this finding in terms of a stronger and/or more efficient focusing of attention on the relevant dimension. One possible implication of this finding could be that binaural gamma beats affect the choice between alternative interpretations of the same stimulus (as in the Navon task) differently than the choice between alternative stimulus events (as in Reedijk et al., 2015), alternative verbal concepts (as in Reedijk et al., 2013), and alternative responses (as in the present task). For instance, focusing visual attention on global features relies on information from different frequency channels than focusing on local features (Hills and Lewis, 2009) and it might be impossible to process both kinds of information at the same time. Another possibility is that a less pronounced global-precedence effect actually represents a broader distribution of resources rather than more focusing. Global precedence might reflect an unequal distribution of attentional resources to the benefit of global information (Robertson, 1996), a rather strong focus that is, so that a reduction of the precedence effect reflects a more equal distribution. If so, the findings from the Navon task would fit reasonably well with our flexibility hypothesis. In any case, the question whether the flexibility hypothesis also holds for the processing and selective attention to global and local features of visual stimuli requires further study.

More research will also be needed to better understand the neural mechanisms underlying both the perceptual illusion that binaural beats induce and the way they affect cognitive-control states. The impact of auditory stimulation on cognitive control is unlikely to be a result of local cortical priming or interactions but rather seems to point neural communication at a larger scale. Larger-scale neural communication has been argued to rely on brain rhythms (Fries, 2009; Brunet et al., 2014), which might be sensitive to binaural beats of particular frequency bands. Indeed, recent studies have shown that beat stimulation affects functional brain connectivity (Gao et al., 2014) and modulates intracranial power and phase synchronization (Becher et al., 2015). These findings support the idea that the impact of binaural beats on cognitive processes might be mediated by neural phase locking (Karino et al., 2006; see, Chaieb et al., 2015, for a recent review on the effect of binaural beats on cognition and mood), in the sense that the beats induce or entrain a particular neural pattern that promotes or impairs neural communication underlying particular cognitive processes, such as cognitive control. Hence, binaural beats may act as a neural entrainment technique that operates by modulating the brain oscillations that particular cognitive processes require or benefit from, and oscillations in the gamma-frequency band might be particularly relevant for this purpose (Pastor et al., 2002; Schwarz and Taylor, 2005). To test that, future studies may make use of electro- or magneto-encephalographic methods, which would permit assessing the relationship between binaural beats and the auditory entrainment of brain oscillations (e.g., Galambos et al., 1981; Picton et al., 1987) and the role of oscillations in the gamma range for local brain communication (Kopell et al., 2000; Quilichini et al., 2010) more directly. Pharmacological studies would also be useful to test, for instance, the possibility that binaural beats involve norepinephrine/glutamate dynamics and increase phasic norepinephrine to enhance cognitive processing (Mather et al., 2015).

In any case, our findings suggest three main conclusions. First, they provide converging evidence for the idea that the current metacontrol state, which we argue implements a particular degree of persistence versus flexibility of cognitive control, can be systematically biased. This supports the general idea that control processes can vary in style (e.g., Goschke, 2003; Cools and D’Esposito, 2011; Hommel, 2015) and the assumption that inducing particular internal states provides an effective means to promote particular styles (e.g., Dreisbach and Goschke, 2004). Second, our findings provide converging evidence for the idea that binaural beats in the gamma range have an impact on the current metacontrol state. While the functional and neural mechanism underlying this impact is not yet entirely understood, the empirical link between the processing of rather low-level auditory stimuli and broadly operating control processes provides rather strong constraints on how this mechanism might work. The question how binaural beats affect brain rhythms related to cognitive control might be key in getting more insight on this issue. Third, together with our previous observations (Reedijk et al., 2013, 2015), the present findings point to some interesting commonalities of, and functional overlap between the selection and consolidation of successive visual stimuli, the sequential search of verbal stimuli in memory, and the separation of sequentially performed tasks. These commonalities seem to support Hommel’s (2015) claim that metacontrol states operate on (i) the degree to which alternative representations compete with each other and (ii) the degree to which their mutual competition is top-down biased through the current goal. In particular, a tendency toward persistence would imply strong competition and top-down bias while a tendency toward flexibility would imply weak competition and top-down bias. If we assume that gamma beats reduce competition and top-down bias, this would explain why processing the second of two targets is less hampered by the first (Reedijk et al., 2015), why searching for multiple words related to the same concept is easier (Reedijk et al., 2013), and why response representations belonging to two different tasks show more crosstalk, as in the present study. Further studies will be necessary to investigate whether and to what degree the biasing of metacontrol states can affect not only the crosstalk between two tasks but also the efficiency to which they can be performed.

In the present study, we found crosstalk effects but no impact of binaural beats on the SOA effect on R2, which is considered to diagnose the bottleneck underlying multitasking. On the one hand, this dissociation between crosstalk and multitasking effects might be taken to challenge the claim that multitasking costs reflect inter-task crosstalk (Navon and Miller, 1987). On the other hand, however, it is still possible that the bottleneck underlying multitasking costs is functional, rather than structural, in nature and that the respective serial processing style is chosen to minimize crosstalk (Miller et al., 2009). In fact, it is possible that increasing crosstalk provides even stronger motivation to serialize as many (other) processes as possible, even though the size of our crosstalk effect might have been too small to make that visible in the SOA effect. To investigate these possibilities more systematically, it would seem to make sense to choose more powerful manipulations to target metacontrol states than those provided by binaural beats, but we leave that to future studies.

## Author Contributions

Authors LC and BH designed the study and wrote the protocol. Authors RS, RF, and SB managed the literature searches and analyses. Author SB collected the data. Authors LC, BH, and RS wrote the first draft of the manuscript. All authors contributed to and have approved the final manuscript.

## Conflict of Interest Statement

The authors declare that the research was conducted in the absence of any commercial or financial relationships that could be construed as a potential conflict of interest.
